# Calls from Beyond the Walls: prison cellphone recordings during the pandemic in Lebanon

**DOI:** 10.1177/01634437221146889

**Published:** 2023-01-13

**Authors:** Chafic Tony Najem

**Affiliations:** Stockholm University, Sweden

**Keywords:** digital technology, media activism, media practice, media witnessing, prisons, Lebanon

## Abstract

Forcibly confined in a precarious and overcrowded space amidst the spread of the COVID-19 pandemic, prisoners in Lebanon resorted to their smuggled cellphones. They produced and circulated images, videos, and sound bites documenting the dire experiences of living under a failing infrastructure. This article addresses this phenomenon by examining a corpus of ‘prison cellphone recordings’ mediated on social media platforms and Lebanese local news. I adopt the media as practice theory to claim that such fragmentary amateur cellphone media messages are the product of strategic and hybrid prison media practices. In addition, I employ the conceptual notions of hybrid media activism and media witnessing to investigate the political and testimonial function of prisoners’ illicit engagement with digital technologies. I propose a typology of the mediated prison cellphone recordings and argue that these representations serve to mobilize support and relay visual evidence of prisoners lived experiences during the pandemic. Finally, I attempt with this article to instigate an approach to the examination of media from the prison; an approach that prioritizes illicit media practices behind bars and their ‘traces’ in the media.

‘We have endured enough!,’ a prisoner declares to the camera of a smuggled cellphone inside Roumieh Central Prison, the biggest and most notorious prison in Lebanon. Surrounded by a group of fellow inmates, he describes in a video the prison’s dire conditions and failed infrastructure amidst the COVID-19 pandemic. News outlets and government officials have reported on the spread of smuggled digital technologies in Lebanese prisons (Najem, 2016). Prisoners in Lebanon have managed to smuggle cellphones, acquire internet connection, circulate media content, and communicate with affiliates and journalists outside the prison. This is deemed illegal and prohibited by Lebanese authorities, and constant efforts are being made to limit the access prisoners’ have to this digital technology (El Hindi, 2013). In this article, I address the illicit digital media use by prisoners in Lebanon by examining, what I elect to term, ‘prison cellphone recordings’ mediated in news and social media and the capacity for such recordings to testify to prisoners’ lived experience of incarceration during the pandemic.

In March 2020, the Lebanese government announced a nationwide lockdown followed by a health state of emergency due to the international spread of the COVID-19 pandemic. However, Lebanese authorities neglected prisoners’ critical situation and ignored the potential crisis the pandemic can cause to the extremely overcrowded penal institutions (Saghieh, 2021). According to Amnesty International (2020), the Lebanese government’s lack of appropriate measures to the spread of COVID-19 in prisons have exacerbated prisoners’ conditions amidst an already dire and failing infrastructure. Accordingly, prisoners mobilized, most prominently in Roumieh Central Prison (RCP). They rioted, protested the unjust treatment, and some declared open hunger strikes (Azhari, 2020). Such mobilizations accompanied an upsurge number of images and videos produced and circulated from behind bars through which prisoners adopted an unprecedented narrative and practices of filming, as I further describe in this article.

By focusing on Lebanon as a case study, I address in this article prisoners’ illicit use of smuggled digital technology during the pandemic. I examine the composition of a corpus of prison cellphone recordings collected from news and social media. Here, prison cellphone recordings are defined as videos, images, and sound bites, captured, produced, and circulated by incarcerated subjects through the illicit use of smuggled cellphones, and the access to internet and telecommunication connection. Then, I propose a typology based on the compositions of such mediated prison cellphone recordings; the *reactive*, *evidential*, and *confrontational type of recordings*. I adopt the theory of media as practice as a conceptual backbone to help me trace the media practices responsible for such representations emerging from the prison. In addition, I employ the notions of hybrid media activism (Treré, 2018) and media witnessing in the aim to reflect on the political and testimonial function of the prison cellphone recordings and the media practices responsible for their production and mediation. I ask, what can such recordings testify to prisoners’ lived experiences amidst the global pandemic? And, how did prisoners utilize media technology to mobilize their content throughout the hybridity of media ecologies?

I conclude that, prison cellphone recordings are the result of hybrid prison media practices aimed to actively mobilize support and affinity. By employing digital technologies, prisoners produce and mediate visual evidence of their lived experiences behind bars. Evidently, certain aspects of this phenomenon remain ambiguous and necessitate further investigation, such as the processes of smuggling, access, and circulation. However, I attempt with this article to shed light on prisoners’ operationalization of media technology amidst the pandemic. As well as I intend to instigate a discussion on the possibilities to comprehend and appreciate a, hidden and unattainable, media practice through the examination of its ‘traces’ in the media. This effort aims to propose an approach to the study of media *from* the prison.

## Media *on*, *in*, and *from* the prison

Correction facilities often exist on the periphery of society and the only perception constructed around them is shaped by the media (Jewkes, 2002). With the growing demand for media coverage and representations of topics of incarceration and crime, the interconnection between the cultural industries and the logic of punishment became sturdier than ever (Brown, 2009; Cheliotis, 2010; Kaun and Stiernstedt, 2020a; Stiernstedt and Kaun, 2021). I can note two trends to the contemporary study of media and prisons in relation to the context of this paper; those focusing on media *on* the prison, and those focusing on media *in* the prison. The first addresses the interconnection between the culture of penal institutions, media industries, and forms of representation (Barton and Brown, 2015; Brown, 2009; Cheliotis, 2010). Adopting a symbolic analysis to images and videos, such scholarship examines the representations and visual culture on and around incarceration, punishment, and prisoners. Topics would range from news representations to surveillance control and technology; most notable to this trend is the emerging scholarship in the discipline of visual criminology (Hayward, 2009; Mawby, 2009).

The second school of thought takes a critical approach to the study of media materiality and technologies inside prisons (Bonini and Perrotta, 2007; Kaun and Stiernstedt, 2020a, 2020b; Stiernstedt and Kaun, 2021). Such scholarship demonstrates that there is a correlation between prisons and media technologies worthy of exploration, such as prisoners’ engagement with radios in prison (Bonini and Perrotta, 2007). As necessary as this scholarship is to understanding prisoners’ quotidian engagement with media technologies, the function and significance of this engagement remains confined within the prison. Further studies on prison and media have proposed the notion of ‘prison media work’ (Kaun and Stiernstedt, 2020a, 2020b; Stiernstedt and Kaun, 2021). Stiernstedt and Kaun (2021) referred to ‘prison media work’ as the administered labor of prisoners in incarceration and their contribution to the vast media and communication industry and cultural production. This seminal historical and materialist analysis to the study of ‘prison media’ traces prisoners’ impact on the development of media technologies and infrastructures at large. Their labor *in* the ‘smart’ prison have contributed valuable data to processes of automation, tracking, surveillance, and data collection (Kaun and Stiernstedt, 2020b: 1288). However, even though such contributions originate from the prison, they are administered by prison administrations and adhere to frameworks and guidelines controlled by governments and media institutions (Kaun and Stiernstedt, 2020b).

I’m proposing in this article a shift toward the engagement with media *from* the prison, which entails the consideration of prisoners’ engagement with a non-institutional or governmental administered forms of media practices resulting in the mediation of media messages to the public. Due to their illicit nature, prison media practices can possibly reflect prisoners’ agency and autonomy in employing media technologies unhinged by the authority’s influence or control. This is not to say that such practices are egalitarian; the cellphone remains a commodity, or even a luxury, acquired by those who can afford it. However, my approach engages with the employment of such technologies as subversive reactions to the controlled environment of incarceration. Investigating media from the prison prioritizes the political potential of the emerged and circulated media messages and the impact they might have in communicating prisoners’ experiences, mobilizations, and testimonies. Therefore, such an approach necessitates a consideration of the illicit media practices responsible for smuggling, accessing, and utilizing digital technologies. It accounts for the processes of mediation, the material technologies, such as that of the cellphone and 3/4 g technology responsible for both producing and circulating the (moving) images, as well as the images and videos themselves and the role they play within various media ecologies.

Naturally, the illicit nature of the phenomenon under study posits a handful of limitations. Questions of access, smuggling, spread of the digital technology behind bars, and the processes of circulation of recordings from the prison to the outside remain in need of further investigation. However, I propose in this article a starting point by collecting and examining the most tangible aspect of this phenomenon; the mediated prison cellphone recordings that are accessible to the public.

## Media as a practice of witnessing and activism

Testimonial and political practices in incarceration, especially those conceived in Arab prisons, have long preceded the smuggling of digital technology. When discussing the digital technological influence on such practices, it is significant to remain attentive of technological determinism and the fascination with material technology (Gerbaudo and Treré, 2015). As well as it is essential to take into consideration the role of prisoners in producing and recordings while also attempting to steer the analysis beyond the mere symbolic examination of media representations. I examine the compositions of the prison cellphone recordings as well as the traces of media practices embedded in such recordings. Adopting the media practice approach is key to this endeavor; it helps in positioning the prisoner as the focal point of this media phenomenon and considers them the main producer of meaning. More specific to the research is the theorization of ‘activist media practices’ (Mattoni, 2012), defined as ‘routinized and creative social practices in which activists engage.’ Activists’ engagement includes both the interactions with media objects and media subjects. The first entails their engagement through the cellphone as they generate and/or appropriate media messages, and the second entails their interactions with media practitioners, such as journalists and social media activists (Mattoni and Treré, 2014: 259). Mattoni (2012) conceptualization of activist media practices is eminent in its approach to media as objects and messages simultaneously, and in its recognition of the subjects behind that media; those who interact, produce, and consume media messages on a systemic and continuous bases (Mattoni and Treré, 2014). This is seminal to the consideration of prisoners as active participants within the production and reception of media messages, while accounting for the specificity of the cellphone and internet as digital media technologies.

Also, the concept of mediation is key in bridging prisoners’ media practices to the flow of media productions, circulation, interpretation, and re-circulation (Couldry, 2008). Mediation within the context of illicit prison media practices benefits the ‘exploration of everyday practices of media appropriation through which social actors enact resistance and resilience to domination and hegemony’ (Mattoni and Treré, 2014: 260). In this essence, mediation should account for the circular process of media production from the prison, the (re)-circulation of media messages through social and news media, the reception of these very media messages by prisoners themselves, and hence, (re) producing messages in relation to this exchange of multiple meanings (Martín-Barbero, 2006).

As the extensive literature on media witnessing have argued, cellphone and digital technology open an opportunity for a witness to channel their testimony during the very act of witnessing, which can help in creating witnesses to distance suffering (Andén-Papadopoulos, 2014; Chouliaraki, 2015; Frosh and Pinchevski, 2009; Torchin, 2012). Given that visitors and journalists were denied access to the prisons during the pandemic, prisoners’ active engagement with digital technology and the recordings they produced and circulated can potentially testify to the otherwise undocumented experiences in incarceration. This is best understood through the notion of media witnessing as ‘witnessing performed in, by, and through the media’ (Frosh and Pinchevski, 2009). Andén-Papadopoulos (2014) argues that citizens’ phone camera recordings have emerged as a ritual of bearing witness, they create visual evidence in the aim to ‘produce and sustain feelings of political affinity and solidarity’ (p. 765). Digital media witnessing becomes ‘an act of representation that publicizes conflict death from the locals’ perspective so as to mobilize emotion and invite a response, be this revenge, outrage, contempt, fear or empathy’ (Chouliaraki, 2015: 1362).

Furthermore, and once produced and circulated by prisoners, prison cellphone recordings get immediately appropriated and re-mediated within news and social media. Re-mediation is seen here as a ‘practice of representation that re-constitutes mediatized death as an authentic event worthy of ‘our’ emotion at the moment that it claims to simply re-disseminate it’ (Chouliaraki, 2015: 1363). With such processes of re-mediation, prison cellphone recordings start to navigate within a hybrid of media ecologies and prisoners, and their media practices, are active actors within such processes. Treré’s (2018) hybrid media activism is a necessary notion here for scrutinizing the hybridity of media technologies at play. According to Treré (2018), activists often employ a complex and unpredictable hybrid of old/new, physical/digital, human/non-human, corporate/alternative technologies to counter, amongst other things, dominant media and state actors while reproducing and strengthening collective visions and identities. This approach to media activism, that of Treré (2018), help demarcate how prison cellphone recordings are employed on social media sites by social movements and prisoners’ affiliates on the outside.

## Methodology: visual traces of media practices

The following research focuses primarily on Lebanon, its incarceration system and its media ecologies. To understand the chaotic nature of phenomena such as the smuggling of cellphones in Lebanese prisons, it is key to briefly consider the historical and socio-political factors which shaped and molded the neo-colonial and sectarian Lebanese system. Amel (1986) model of sectarian hegemony critically dissects the ruling arrangement of the Lebanese sectarian model and the emergence of sectarian identities and partitions. Amel (1986) argues that the bourgeois rule in Lebanon necessitated forms of sectarian and class representation as a result of a power sharing consensus between the financial class and partisan political leaders. This has prominently influenced the corruption within the judicial system, penal institutions, the administration and seclusion of prisoners, as well as the Lebanese media sphere^1^ (Najem, 2016).

Prisons in Lebanon suffer from failed infrastructure, miserable conditions, overcrowding, arbitrary arrests, and segregation of prisoners based on partisan and sectarian ideologies (El Hindi, 2013; Nashabe, 2003). Partisan sectarian identities have further influenced the fragmentations and affluence behind bars. As prisoners from different partisan partitions have produced prison cellphone recordings, it becomes imperative to investigate the illicit use of media technologies in an environment where the exchange of information is heavily controlled and prohibited. RCP is the central, biggest, and most notorious incarceration institution in Lebanon. It hosts a selection of individuals across the criminological spectrum; minor and major offenders, people with terrorism charges, and individuals still awaiting their prosecution (El Hindi, 2013; Nashabe, 2003). RCP consists of various block buildings according to which prisoners are divided. Bloc B, an infamous building in RCP, hosts individuals incarcerated on terrorism charges and affiliation with fundamental Sunni groups; they are often referred to as *Islamists* in local and international media (Lons, 2016). Bloc B has a history of violence with the authorities and is reported to have smuggled digital technologies and internet access due to its close connections with powerful fundamentalist figures (El Hindi, 2013).

The corpus of this study consists of images and videos produced illicitly by prisoners and circulated on social media, YouTube and Facebook, and appropriated by local Lebanese TV news reports. The process of data collection was as follows; I monitored local and international news reports covering the outbreak of COVID-19 virus in Lebanese prisons from March 2020, when the first three cases in Lebanon were detected, until March 2021. Furthermore, I conducted a search on Facebook and YouTube based on three main hashtags in Arabic; ‘Roumieh Prison,’ ‘Lebanese Prisons,’ and ‘Corona in Prison.’ I collected the images and videos resulting from this search spanning from March 2020 to March 2021. News reports, both local and international, have used snippets of cellphone prison recordings in their reports on the prison. On Facebook, the recordings were mostly uploaded by Facebook pages in support of prisoners, mainly those supporting Islamist prisoners. And, on YouTube, videos were uploaded by online news platforms and anonymous users, the first were edited videos while the second were small snippets of ‘raw’ footage from the prison. According to AP News, the content reached an exponential peak in September 2020 in response to over 200 detected cases of COVID-19 in RCP (Mroue, 2020).

The analysis was conducted according to Rose (2016) framework of Critical Visual Methodology, while focusing primarily on the site of the image and that of production. I analyzed the content following six analytical tools for the analysis of the collected corpus of material; the composition, POV, frame, montage, sound, (re)mediation, and the sectarian socio-political context and colonial history of Lebanon. The purpose of these analytical tools is to move the analysis beyond just the symbolic analysis of representation and attempt to understand the practice of video and image production behind bars through the visual traces. Each analytical tool in this case can be delineated visually on the screen while still reflecting the practice responsible for it. Hence, this creates a connection between the images and videos, the material practices implemented by prisoners, the context of the spread of COVID-19 within the precarious and harrowing prisons in Lebanon, and the narratives constructed by news reports through the re-mediation of prison cellphone recordings.

## Prison cellphone recordings – three types

After the examination of the collected corpus of material, and through the aforementioned analytical tools, I can categorize three main types of prison cellphone recordings produced and mediated by prisoners during the pandemic: *reactive*, *evidential*, and *confrontational*. The first, the *reactive* type, is more common within leaked footage of violent events, whereas the *evidential* and *confrontational* seem to be newly emerging with the spread of the virus behind bars.

**Figure 1.**
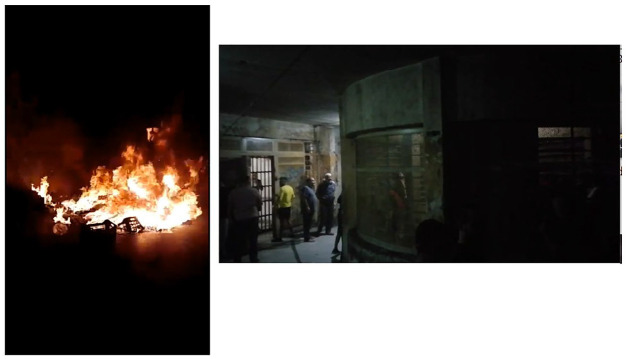
Mobilizations in Roumieh Central Prison recorded through prisoners’ cellphones.

The *reactive* type comprises images and videos of riots, violence, and mutiny in response to the governments’ measures, or lack thereof, amidst the vast spread of COVID-19 in prisons (Figure 1). Recorded with fast and unfocused camera movements, the prisoner shoots from his POV to capture the overwhelming scenes of mutiny, screaming, and destruction. Content is often dim and lacks verbal contextualization, comprehensible dialog, or narration. This low resolution and fumbled frame seem to be a product of prisoners’ immediate reaction to documenting the overwhelming violent events as they unfold, hence the term *reactive*. This type of recordings can be approached as ‘an act of representation that publicizes conflict death from the locals’ perspective so as to mobilize emotion and invite a response, be this revenge, outrage, contempt, fear or empathy’ (Chouliaraki, 2015: 1362).

In addition, recordings of this nature aim to be sensible more than intelligible (Andén-Papadopoulos, 2014). Otherwise, any additional information contextualizing these recordings is provided by the party remediating the content, such as a TV news report or a Facebook page. The reactive recordings speak to the four ‘aesthetics of authenticity in citizen imagery’ during precarious times listed by Andén-Papadopoulos (2014); hypermobility, opacity, non-narrativity, and ‘raw’ audio. The aimless camera movements, the dim environment filmed, the overwhelming ‘raw’ sounds of screaming, and the lack of contextualization render the reactive recordings hypermobile, blurry, and non-narrative, hence confronting us with ‘an uncertain form of representation that oscillates between figure and abstraction, transparency and opacity’ (Andén-Papadopoulos, 2014: 346).

**Figure 2.**
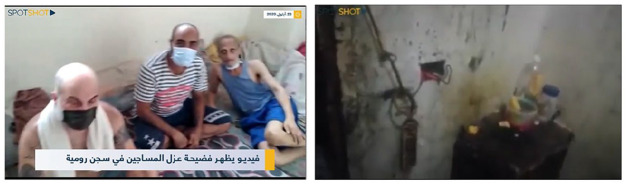
Cellphone video footage of infected prisoners and dire conditions. Recorded and circulated from RCP, the video is remediated by a Lebanese online news platform.

The *evidential* type comprises recordings documenting the prison’s dire conditions and failed infrastructure, such as the waste on the floor, the unsanitary environments, and the congested spaces with bodies (Figure 2). It is often produced with the intention to create tangible evidence or proof of the experiences during the pandemic, hence the term *evidential*. From their POV, prisoners use the cellphone camera to record their environment, fellow inmates, or even the authority personnel. Evidential recordings are longer in format and often contain a voice-over by the prisoner contextualizing a specific situation as the latter ensures to include elements necessary for their narrative. If they are referring to the lack of sanitation, they focus the camera on the corners full of waste and debris. When they relay the suffering of their fellow inmates, they make sure to capture the vulnerability of the bodies and faces of individuals infected with the virus. The prisoners’ engagement with digital technology results in a well-constructed, almost journalistic, type of recording featuring visual proofs and interviews with fellow inmates supporting their voice over.

In one of the videos examined, the prisoner slowly films the bodies lying on the floor allegedly infected with the virus. The video is mostly calm, unlike the previous example, no yelling or screaming are heard. Instead, it contains sounds of discussions in the vicinity and sporadic coughing of prisoners sleeping on mattresses. ‘This is what is really happening in the confinement,’ the prisoner iterates as he records the bodies on the floor, ‘people are dying and no one is asking about them.’ ‘What is wrong with you?!’ he adds. The camera moves toward a prisoner lying on his back on the floor. ‘I’m sick, I’m sick. . .temperature is high’ he replies with a dimmed voice and a surgical mask around his chin. ‘You didn’t see a doctor?’ the prisoner behind the camera asks, ‘there is no one’ the sick prisoner replies. The conversation continues and the camera phases away from the sick prisoner. ‘This is the confinement, and this is the team of doctors, the team of doctors the ministry of interior affairs sent out yesterday. . .’ the prisoner behind the camera sarcastically adds before the video ends. This video was uploaded by an anonymous account on YouTube, however scenes and snippets from it were remediated by news TV reports and online news blogs. The prisoner, still aiming to ‘produce and sustain feelings of political affinity and solidarity’ (Andén-Papadopoulos, 2014: 765), shifts their practice of filming toward a more comprehensible evidence based content. They frame their recordings as the ‘true reality of Roumieh Prison’ eluding to the idea that every other reality constructed by the authorities is in fact flawed.

**Figure 3.**
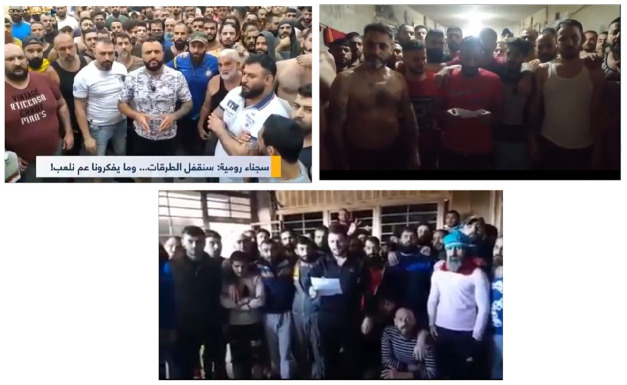
Prisoners in RCP congregate and address the smuggled cellphone camera with their demands and testimonies.

The *confrontational* type comprises a series of performative enactments toward the camera (Figure 3). A group of inmates stand silently looking straight into the cellphone camera filming them, one of them stands in the middle and, clearly and loudly, addresses the audience. Various videos under this type had this same composition which suggests the emergence of a new practice of prison mobilization centered around the cellphone, the camera, and the presupposition that the videos will be mediated. The prisoners take an aggressive position, they stand, stare into the camera, and address the audience, hence the term *confrontational*. They are not rioting against the prison administration, neither they are vulnerable on the floor, instead, they ‘have had enough of this injustice and they are ready to take drastic measures,’ as one of them stated. They do not plead for help, instead they threaten of mutiny; they address politicians and citizens alike.

During some videos, prisoners even tied ropes around their necks and attempted to enact forms of self-killing or they laid down on the floor. No harm was inflicted during the filming of the footage, yet prisoners’ reactions suggested that such self-killing could take place at any moment. Acts of self-harm and self-killing are common practices of resistance behind bars (Bargu, 2014). However, the significance of the cellphone camera here is that it transforms these acts into re-enactments. The prison media practices allowed prisoners to engage in such practices of resistance without having to physically commit the actual act of self-harm. This suggests a development in prison mobilization practices that converge the act of mobilization with the act of filming. The presence of the cellphone, and the media practice it enabled, shape the formation of the mobilization. Prisoners congregate, choose a spokesperson, pick a narrative, address the audience with their vulnerabilities, anger, needs, and demands, and presuppose that the footage recorded will in fact disseminate and reach the public.

## Mediation of prison cellphone recordings and the hybridity of the practice

Based on the cellphone recordings analyzed, prisoners’ recordings represent the suffering and even deaths resulting from the authorities’ negligence in containing the spread of the virus. This is manifested through the images of suffering and the narration and language adopted by prisoners, as well as the active representations of the physical mobilizations of prisoners such as riots, congregation of bodies, and enactments of self-harm. The smuggled cellphone, its camera, and the accessed internet and telecommunication connection, get ritually employed as part of a media practice to create filmed testimonies and a public record of embodied political dissent (Andén-Papadopoulos, 2014; Chouliaraki, 2015). However, mediation, and re-mediation, of prison cellphone recordings through news and social media is far from a transparent process. Lebanon’s media industry is divided along sectarian and partisan divisions. Each Lebanese TV channel is backed by stakeholders influencing both its funding and its partisan narrative in covering local and international events.^2^ Evidently, TV news reports would appropriate prison cellphone recordings during the pandemic within their own political narrative.

For instance, *MTV*, a right wing Christian TV station would report on the precarious conditions of RCP, utilize prisoners’ recordings, then, accuse Islamist prisoners of spreading COVID-19 in the prison. While another TV channel, such as *Al-Jadeed*, a self-proclaimed progressive TV station, would utilize prison cellphone recordings and critique the government’s failure in containing the spread of COVID-19 and in preventing the smuggling of cellphone into prisons. This approach to framing journalistic stories is common in the Lebanese media sphere. However, I argue that during the pandemic, prisoners adopted a hybrid media approach within their media practices; an approach resembling Treré’s (2018) conceptualization of hybrid media activism. They utilized different facets of digital technology, circulated their content through various media ecologies, and took an active part in the mediation, appropriation, re-contextualization of narratives on the prison. Prisoners have mixed between offline and online mobilizations, utilized images, videos, phone calls, voice recordings, and developed practices adhering to the presence of digital technology behind bars, such as those reflected under the confrontational type of recordings.

**Figure 4.**
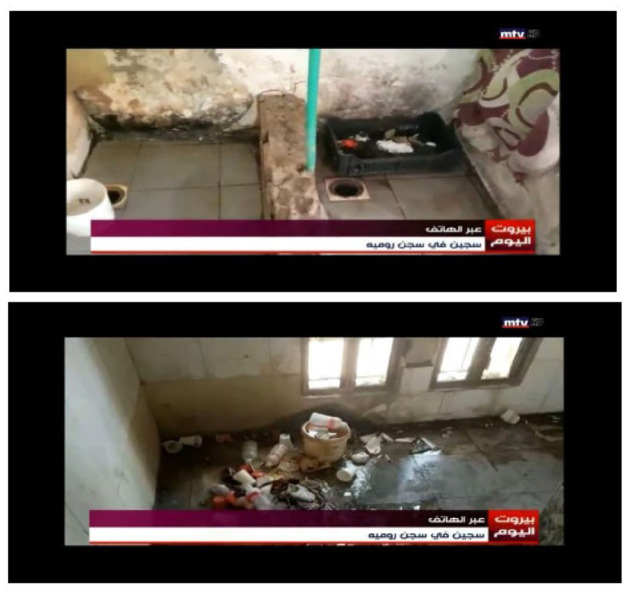
Prison cellphone recordings of dire conditions and failed infrastructure from RCP broadcasted by Lebanese local TV news. Caption reads: ‘on the phone: prisoner from Roumieh prison.’.’

During one of their special reports on the spread of COVID-19 in RCP, a reporter on a TV news program receives a live phone call from a prisoner to inquire about the situation of the prison (Figure 4). The prisoner, through his smuggled cellphone, expresses his appreciation to the reporter’s interest in the matter and moves on to describe the dire conditions and the increasing numbers of infected individuals. The phone call is juxtaposed on the screen by video footage captured through a smuggled cellphone. A hybrid of two testimonies, an audible and a visual, are put together side by side through a TV news report. The hybridity of technologies within the media practice enabled the prisoner to record the footage through the camera, disseminate it through backstage communication networks to reach the reporters,^3^ and conduct a phone call through the telecommunication network of the cell phone. Through hybrid media activism, prisoners attempt to operationalize various media ecologies; they conduct phone calls, take photographs, film testimonies, consume media, and establish communication networks to ensure the circulation of their recordings to either journalists or affiliates on the outside. Hence, prison cellphone recordings are dependent on multi-dimensional media practices aiming to utilize the hybridity of digital technology to relay prison testimonies.

To conclude, prison cellphone recordings can seemingly appear as chaotic and random snippets of media messages circulating within the media sphere. Yet, such recordings reflect prisoners’ ability to develop hybrid forms of prison media practices for the purpose of political affinity. With prisoners’ engagement with digital media technologies, the previously unimaginable ‘reality of prison’ can begin to be partially witnessed by those of us on the outside (Andén-Papadopoulos, 2014; Bonini and Perrotta, 2007: 180). Historically, prisoners have managed to mediate their experiences through mediums such as prison novels or letters (Hafez, 2002). Even though smuggled digital media technologies have facilitated the immediate circulation of media messages, they constitute more than a mere medium for prisoners’ experiences. Digital technologies behind bars contribute to the inception of various media practices; they reflect new strategies of production, circulation, and communication. Prisoners’ use of smuggled technologies could even contribute to the formation of illicit media infrastructures; ones that do not adhere to governments and institutional control as in the case discussed by Kaun and Stiernstedt (2020b).

Moreover, the context of Lebanon demonstrates the precarious and deadly conditions created by neocolonial states and their penal institutions and the subversive practices such oppressive spaces can incite. Forcibly confined under a failing infrastructure, and facing the spread of a global pandemic, prisoners took illicit media practices as recourse. They utilized, interacted, and engaged with smuggled media technologies to spread testimonies of lived experiences of imprisonment. The pandemic, as a temporal event, instigated the production and circulation of prison cellphone recordings from prisons worldwide (Hamilton, 2020). Even though we might not be at a close proximity to conceptualize prisoners’ illicit use of digital technologies during these events, it is still imperative to engage and investigate such practices. In this article, I attempted to do so by tracing the prison media practices through their representations in the media. By doing so, I proposed an approach to the study of media *from* the prison; an approach based on the examination of prison cellphone recordings as an initial step to tracing the illicit media practices behind bars.

## Notes

1. Media institutions in Lebanon often ensure that their news reporting on incarceration and prisoners’ stories fall in the direction of the institution’s partisan ownership (Najem, 2016).

2. See Sarah El-Richani – The Lebanese Media: anatomy of a system in perpetual crisis (2016).

3. Usually such communication networks are established through chat apps, mainly Whatsapp.
